# Reply to the Comment on “Melanisation of *Aspergillus terreus*—Is Butyrolactone I Involved in the Regulation of Both DOPA and DHN Types of Pigments in Submerged Culture? Microorganisms 2017, 5, 22”

**DOI:** 10.3390/microorganisms5030036

**Published:** 2017-07-04

**Authors:** Elina K. Palonen, Sheetal Raina, Annika Brandt, Jussi Meriluoto, Tajalli Keshavarz, Juhani T. Soini

**Affiliations:** 1 Biochemistry, Faculty of Science and Engineering, Åbo Akademi University, Artillerigatan 6, Åbo FI-20520, Finland; annika.brandt@abo.fi (A.B.); jussi.meriluoto@abo.fi (J.M.); 2 Department of Life Sciences, University of Westminster, London W1W 6UW, UK; rainas2@googlemail.com (S.R.); t.keshavarz@westminster.ac.uk (T.K.); 3 Faculty of Life Sciences and Business, Turku University of Applied Sciences, Lemminkäinengatan 30, Åbo FI-20520, Finland; juhani.soini@turkuamk.fi

We are pleased that our paper has generated this discussion. We left the title of our paper with a question mark as we thought that some of the findings we made would require further exploration.

We are inclined to agree on the presented issue. In light of the evidence that you give in your comment, it appears that Asp-melanin is not of DOPA type due to the lack of solvent control in the study of Pal et al. [1]. We appreciate your expertise on this matter. It was not our aim to reject the production of Asp-melanin in conidia during static or biofilm growth conditions nor the novel pathway which were revealed by Geib et al. [2]. We admit that the used inhibitors by Pal et al. [1] may not be specific enough, allowing divergent presumptions of the involved enzymes.

However, the focus of our study was to characterise the revealed NR-PKS gene *pgmA* and the surrounding gene cluster on a transcriptional level by utilizing the obtained gene expression and transcriptome sequence data. We presented only a speculation regarding the function of this cluster as remarked in the comment presented by Geib and Brock [3].

Regarding the several further thoughts presented by Geib and Brock [3], we agree on the necessity to confirm them with further studies. In our previous study [4], we observed an increase in lovastatin production, both on the transcriptional and molecular level, and showed it to be enhanced already 24 h after butyrolactone I supplementation, 96 h prior to the speculated *pgm* cluster genes showed upregulation as described in our current study [5]. Similarly, in our recent study [6], we observed the global regulator gene *laeA* to be upregulated 24 h after butyrolactone I supplementation, i.e., 96 h prior to the speculated *pgm* cluster genes. Furthermore, Bok et al. [7] reported LaeA to regulate lovastatin production in *A. terreus* as well, supporting the hypothesised role of *laeA* and the secondary metabolism occurrence. The speculation regarding the role of *pgm* cluster genes in secondary metabolism is based on these observations.

Naturally, all these questions require further investigations as proposed by Geib and Brock [3]. Schimmel et al. [8] reported butyrolactone I to increase the number of spores as well as lovastatin production. The studies of Raina et al., Palonen et al. and Schimmel et al. [4,5,6,8] were performed in submerged and continuously shaken culture conditions, although we have no specific morphological information regarding sporulation. Therefore, we do agree that none of these observations define the function of this *pgm* gene cluster to be specifically related to pigmentation but a difference with the occurred secondary metabolism was hypothesised.

We can accept that the properties of the *pgm* gene cluster to produce pigment are unconfirmed by biochemical methods. As pointed out by Geib and Brock [3], the observed similarity with the fusarubin gene cluster of the *Fusarium fujikuroi* may indicate sexual development during the growth conditions used in this study. This raises a question regarding the observed upregulation of one of the key regulators of asexual conidiation, *abaA*—occurring exactly at the same time point as the *pgm* cluster genes are upregulated as well [5,6]. Some of the regulative genes of sexual development were also expressed under the applied growth conditions (data not shown). The potential production of accessory conidia cannot be ruled out, but this type of spores is reported to be unpigmented in addition to being hyaline [9].

Taken together, we thank Dr. Geib and Dr. Brock for their careful reading and constructive comments. We hope to have the chance to study the melanisation of *Aspergillus terreus* together in future projects.

## References

[1] Pal A.K., Gajjar D.U., Vasavada A.R. (2014). DOPA and DHN pathway orchestrate melanin synthesis in *Aspergillus* species. Med. Mycol..

[2] Geib E., Gressler M., Viediernikova I., Hillmann F., Jacobsen I.D., Nietzsche S., Hertweck C., Brock M. (2016). A non-canonical melanin biosynthesis pathway protects *Aspergillus terreus* conidia from environmental stress. Cell Chem. Biol..

[3] Geib E., Brock M. (2017). Comment on: “Melanisation of *Aspergillus terreus*-is butyrolactone I involved in the regulation of both DOPA and DHN types of pigments in submerged culture? Microorganisms 2017, 5, 22.”. Microorganisms.

[4] Raina S., de Vizio D., Palonen E.K., Odell M., Brandt A.M., Soini J.T., Keshavarz T. (2012). Is quorum sensing involved in lovastatin production in the filamentous fungus *Aspergillus terreus*?. Process. Biochem..

[5] Palonen E.K., Raina S., Brandt A., Meriluoto J., Keshavarz T., Soini J.T. (2017). Melanisation of *Aspergillus terreus*-is butyrolactone I involved in the regulation of both DOPA and DHN types of pigments in submerged culture?. Microorganisms.

[6] Palonen E.K., Raina S., Brandt A., Meriluoto J., Keshavarz T., Soini J.T. (2017). Transcriptomic complexity of *Aspergillus terreus* velvet gene family under the influence of butyrolactone I. Microorganisms.

[7] Bok J.W., Keller N.P. (2004). LaeA, a regulator of secondary metabolism in *Aspergillus* spp.. Eukaryot. Cell.

[8] Schimmel T.G., Coffman A.D., Parsons S.J. (1998). Effect of butyrolactone I on the producing fungus, *Aspergillus terreus*. Appl. Environ. Microbiol..

[9] Deak E., Wilson S.D., White E., Carr J.H., Balajee S.A. (2009). *Aspergillus terreus* accessory conidia are unique in surface architecture, cell wall composition and germination kinetics. PLoS ONE.

